# The effect of incongruous contextual cues on pictorial metaphor processing

**DOI:** 10.3389/fpsyg.2022.966386

**Published:** 2022-12-20

**Authors:** Yongyi Mo, Rong Zhou, Xi Chen

**Affiliations:** ^1^The Center for Language Cognition and Assessment, School of Foreign Studies, South China Normal University, Guangzhou, China; ^2^School of Psychology, South China Normal University, Guangzhou, China

**Keywords:** perceptual incongruity, conceptual incongruity, context, pictorial metaphor processing, eye-tracking

## Abstract

An eye-tracking experiment was conducted to investigate the effects of incongruous contextual cues, both perceptual and conceptual, on pictorial metaphor processing. In a metaphoricity decision task, 38 participants independently viewed a total of 36 pictures equally divided into three different conditions: metaphoric pictures (MP), anomalous literal pictures (ALP) with perceptual congruity, and congruous literal pictures (LP). By initiating the midway condition ALP, the effect of contextual cues of conceptual incongruity were distinguished from that of perceptual incongruity. The eye movement data during each picture viewing were collected before the participant made a decision whether the picture was metaphorical or not. The behavioral results showed that the more abundant incongruous contextual cues were there, the more likely the pictures would be judged as metaphors. It took longer for the participants to make decisions on the literal pictures, be them in congruous or incongruous condition. The results of eye-tracking statistics showed that the perceptual incongruity cues were detected earlier than the conceptual ones. The perceptual-conceptual incongruity cues evoked more fixations and longer duration than the perceptual incongruity alone. The processing of conceptual incongruity proceeded after that of perceptual incongruity. The overall result of the study supports the contextual cues of perceptual incongruity as triggers for pictorial metaphor processing, whereas the contextual cues of conceptual incongruity play a decisive role in the metaphoric interpretation, which in turn renders the processing of MP more mental effort consuming than that of ALP or LP. The present findings have vital implications in revealing the triggering and determining mechanisms of pictorial metaphor processing, which are significant in exploring human cognition and have great impacts on various facets of social and cultural communications.

## 1 Introduction

As a main way of thinking and conceptualization, metaphors are inherent to the human being and direct consequences of the interaction between our particular physical and cognitive make-up, and our experiences in the world (Grady, 1997). Metaphors are widely used in verbal expressions and frequently presented in pictures as well. A pictorial metaphor is the metaphor generated by the synergy of source domain and target domain of a static picture (Forceville, 2006). The metaphoric interpretation of a picture can be supported by understanding three types of visual structures, namely, “Juxtaposition,” “Fusion,” and “Replacement” (Philips and McQuarrie, 2004). For example, a clock and coins brought together in a picture represents the metaphor TIME IS MONEY. When a man’s body is fused with a shark’s head, the metaphor BOSS IS SHARK may come up vividly for a greedy and ferocious boss. If in a picture someone is watering a plant, with the flower being replaced by a gold coin, then the metaphor INVESTMENT IS GROWING PLANT may occur to the viewer. Pictorial metaphors reflect the way people understand and construct the meaning of abstract ideas. They are important approaches and means for people to know the unknown world and construct novel conceptual schemata. The research into pictorial metaphor processing can effectively testify the non-verbal nature of metaphor (Forceville, 2006). It is thus of significant theoretical value for studying the mental representation of concepts, for establishing the relationship between visual and semantic processing, and for revealing the mechanism of visual processing in the brain. Furthermore, studies of pictorial metaphor are of more advanced pragmatic value, for artificial intelligence may tap from the metaphorical approach only possessed by humankind to recognize more complex images.

Pictorial metaphors are often dynamic, irregular, and even novel, with the context being an essential element in the generation of metaphoric meaning (Kövecses, 2015, 2020). If a metaphor is primarily a matter of thought rather than a matter of language (Lakoff and Johnson, 1980), then metaphorical processing mechanisms should be the same for the interpretation of both verbal and pictorial metaphors (Cavazzana and Bolognesi, 2020). Based on Conceptual Metaphor Theory (Lakoff and Johnson, 1980) and the Conceptual Integration Theory (Fauconnier and Turner, 2002), metaphor processing mainly relies on the cross-domain interactive mapping and conceptual integration of the two input spaces. Because of the particularity of pictures, such processing also involves the selection of specific representations of the source domain and the target domain in metaphorical relations, i.e., perceptual (e.g., shape) representations and conceptual (e.g., function) representations (Gernsbacher and Robertson, 1999). The reasoning process of visual metaphor comprehension involves “the mutual cognitive environment, or, the context” (Kövecses, 2015:178). Van Dijk (2009) postulates that contextual content is represented by the conceptualizers as a “context model.” A context model is ideally a cognitive model of the situation in which communication takes place that comprises a number of components, including: Setting (time, location, circumstances, props), Happening (actors and various relations, personally, socially, mentally), and Activity/Conduct (ibid.: 39). In a pictorial metaphor, “dominantly literally conceived source (domain)” (Kövecses, 2015:119) constituted the context of a picture. The various contextual cues can “motivate, trigger, prompt, facilitate, shape, etc., the use of a metaphor in discourse” (ibid, 191). In the complex meaning network of contextual cues, each node can be a potential meaning trigger (Taylor, 1995; Zhou, 2011), which can guide or prompt the global meaning and hence can help people identify the symbol, the reference, the metaphor and other meanings of a picture. In metaphor processing, contextual cues can effectively activate the cognitive framework of the anchored meaning, extracting the conceptual nodes attached to it while eliminating or suppressing inappropriate meaning, so as to access or strengthen the most relevant metaphorical meaning in accordance with the context.

In this study, we focus on one kind of contextual cues—contextual incongruity, for it is considered a necessary condition for identifying novel metaphors (Romero and Soria, 2013). According to previous research into visual metaphor advertisement processing, viewers would “use deviation from expectation as a cue to start thinking about possible metaphorical interpretations” (Steen, 2018:134). In the present study, incongruity is regarded as contextual cues, which refer to “some of the techniques by which the viewer’s understanding of physical reality can be violated” (ibid., 57), such as: modification of physical characteristics, inappropriate setting or depicted function, and juxtaposition (Kaplan, 2005). Contextual incongruity appears when the literal interpretation of a picture is inconsistent with the given context (Ortony, 1980). Perceptual incongruity such as deformation, distortion, and dislocation, etc., will inevitably emerge, as the entities belonging to the source domain and those belonging to the target domain are forced into the same picture, which is dubbed by Carroll (1994) as *homospatiality*. Therefore, in a metaphoric picture (MP) there usually exist two kinds of incongruous contextual cues: one perceptual and the other, conceptual. Perceptual incongruity refers to the conflict at a physical level where the color, contour, shape, texture, orientation, and other visual expression of an object do not conform to their expected features in the real world (Yang, 2013). Conceptual incongruity refers to the conflict at a semantic level that violates the restriction of semantic choice or common sense in the pictorial contextual complex. Take a pictorial metaphor from the “Replacement” category for example (see Figure 2 in Section “2.3 Procedure and data analyses”). The picture depicts a person writing with a pen in his hand, but in the original place of his brain was a paper wad instead. Within the ordinary visual context of writing and thinking, the paper wad in the man’s head constitutes both perceptual incongruity (the visual conflict between the perception or shape of the brain and that of the paper wad), and conceptual incongruity (the conflict between the conception or function of the brain and that of the paper wad). If the corresponding position in the picture was a human brain rather than a paper wad, it could be literally interpreted as “*man is thinking*.” However, the visual anomaly with a paper wad taking the place of the brain formed the incongruous contextual cue “*brain is paper wad*,” which may prompt the viewer to interpret the picture *via* a non-literal approach, and ultimately deduce the metaphoric meaning, such as “*a tangled mind*” or “*futile thoughts*.” Such “Replacement” metaphors are also known as *contextual metaphors* because they are highly dependent on the pictorial context for meaning construction (Forceville, 2016). In a contextual metaphor, a visually rendered object is turned into the target of a metaphor by being depicted in a visual context in such a way that the object is presented as if it were something else—the source (ibid.). It is the visual context that provides the source. Ortony (1980) once postulated that a first requirement for something to be a metaphor is that it should be contextually anomalous, which means that a literal interpretation fails to fit the context. In the literature, contextual incongruity has been addressed under different names, for example, “contextual anomaly” (Ortony, 1980), “contextual abnormality” (Romero and Soria, 2013), “conceptual incongruity” (Kittay, 1987), or simply “incongruity” (Dynel, 2011), and “anomaly” (Romero and Soria, 2013). In this paper, we use “contextual incongruity” to refer to the above terms, which can be defined as the anomaly appearing in certain unit(s) of a picture due to the perceptual or conceptual mismatch(es) between the entities depicted in the picture. Schilperoord (2018) emphasized that contextual incongruity can be regarded as an independent property and necessary feature of pictorial metaphors, while Romero and Soria (2013) took it as a necessary but not sufficient condition for metaphors. It is the visual representation of those incongruities that forces the viewer to make inference about the relationship between the source domain and the target domain (Carroll, 1994).

Perceptual and conceptual processes are two qualitatively different psychological processes in operation at two consecutive times, with an interval of roughly 300 ms dividing them (Loftus and Ginn, 1984). Perceptual similarity between a source domain object and a target domain object, especially similarity in shape, can facilitate metaphor processing (Cao et al., 2019; Ojha and Indurkhya, 2020), and further enhance the conceptual link (Van Weelden et al., 2011). Indurkhya and Ojha (2013) used pictorial metaphors of the “Juxtaposition” category in an eye-tracking experiment and found that the detection of perceptual similarity occurred at the pre-attentive level. This finding helped to explain why incongruity had not been reported by the participants when a thinking-aloud paradigm was adopted (Steen, 2018). However, previous studies did find that metaphor processing induced a larger Event-related Potentials (ERP) amplitude in the early time window, which may have been caused by the semantic anomaly of the picture (Ma et al., 2016; Ortiz et al., 2017). Some other studies have found that contextual incongruity, either perceptually or conceptually, can facilitate metaphorical processing (Van Weelden et al., 2011; Hu et al., 2014; Cao et al., 2019), but some others, in contrast, have found contextual incongruity inhibiting metaphorical comprehension (Inhoff et al., 1984; Heckler and Childers, 1992; Boujena et al., 2021). These seemingly contradictory conclusion may have been caused by different research methodologies and materials, or by mingling different stages of metaphor processing. Therefore, the role of incongruous contextual cues in metaphor processing remains unclear. Besides, previous studies on metaphor processing were mainly focused on linguistic metaphors, some of which had mixed together such factors as the context and familiarity (Pynte et al., 1996), and some of which only had single linguistic cues, instead of more authentic and complex contextual cues (Li et al., 2020). The value of research on pictorial metaphor processing lies in that it can effectively distinguish between lexical semantics and conceptual structures (Steen, 2011). However, most of the previous studies on pictorial metaphor processing have only manipulated pictures of single objects without contextual cue (Van Weelden et al., 2011; Hu et al., 2014; Ma et al., 2016; Cao et al., 2019), so much so that the function of contextual cue is still unclear. Some studies have actually used novel MP with authentic and complex context in the experiments, and also have discussed the effect of incongruity on metaphor processing (Indurkhya and Ojha, 2013; Ortiz et al., 2017), however, the distinct effects of perceptual incongruity and conceptual incongruity on metaphor processing have remained unexplored. In view of the above analysis, questions should be further explored concerning when and where an individual detects the contextual cues of incongruity in the picture, and how one uses these cues for cognitive manipulation in order to access the intended meaning by metaphor processing. For this end, eye-tracking technique can be used due to its authenticity, non-intrusiveness and moment-to-moment data source. In order to compare with the baseline—the literal picture (LP) without incongruity, apart from the MP with both perceptual and conceptual incongruity, we have also included a “midway” condition between the metaphoric and the literal—the anomalous literal picture (ALP), which merely has perceptual incongruity. We have collected the temporal and spatial data in the eye-tracking experiment while the participants were viewing the three different types of pictures. Our purpose is to discover the cognitive pattern and mechanism of pictorial metaphor processing. Besides, the effects of incongruous contextual cues can be further differentiated into perceptual and conceptual. To that end, we propose three research questions: (1) Do incongruous contextual cues influence pictorial metaphor comprehension? (2) What’s the trigger of metaphor processing, perceptual incongruity or conceptual incongruity? and (3) How do perceptual and conceptual incongruous contextual cues affect the difficulty in processing pictorial metaphors?

In the present experiment, metaphoricity and incongruency are two main factors that influence the picture processing. Metaphoricity is “a scalar value” (Dunn, 2014:746) measuring the fact or quality of being metaphoric, ranging from metaphoric to literal. Incongruency is separated into overall incongruency and two subscales: perceptual incongruency and conceptual incongruency. Overall incongruency means the overall degree to which certain incongruities, or anomalies caused by a literal reading, may violate the viewer’s understanding of reality (Schilperoord, 2018:16). “Perceptual incongruency” refers to the degree of incongruity to which pictorial elements seem distorted or out of place, or other anomalies that violate the viewer’s understanding of physical reality. “Conceptual incongruency” refers to the degree of incongruity to which the source domain conflicts with the target domain. Apart from metaphoricity and incongruity, complexity, and difficulty are two more factors to be controlled as they may influence the result of our experiment. “Complexity” here incorporates visual complexity and structural complexity, which measure whether the pictures contain dense perceptual features and/or an elaborate design complexity of the objects, such as quantity, details, asymmetry, or irregularity of arrangement, etc. (see Pieters et al., 2010 for details). “Difficulty” refers to the degree to which the individual feels it difficult to comprehend the meaning of the given picture.

## 2 Materials and methods

### 2.1 Participants

Forty-two undergraduate and post-graduate students (23 women, 19 men) from different majors of South China Normal University voluntarily participated in the study. The participants were recruited using convenience sampling. All participants were right-handed, with an average age of 23 ± 2.02, with normal or corrected-to-normal vision. Informed consent was obtained from all participants before the experiment and they were paid after the experiment. Data from four participants were discarded because of bad records probably due to head movement or squinting during the experiment.

### 2.2 Materials

The materials used in the experiment consisted of three sets of pictures: MP (metaphoric pictures with both perceptual and conceptual incongruity), ALP (anomalous literal pictures with only perceptual incongruity), and LP (literal pictures without any incongruity). MP are all contextual metaphors, that is, in a familiar scene (*usu*. of the source domain), a target domain object has taken the usual place of a source domain object. Thus in MP, there overtly exist contextual incongruous cues both perceptually and conceptually. ALP only depicts objects in a single conceptual domain, but the visual elements (e.g., shape, size, line, texture, or position) of a certain object in the picture are conflicting with those in reality (Yang, 2013), so that ALP only has perceptually incongruous contextual cues. LP also presents things in a single conceptual domain, but without contextual incongruity, as everything in the picture is in accordance with real life. Twelve pictures for each set were selected from an illustration reservoir on an Internet image website^1^ (see Figure 2 in Section “2.3 Procedure and data analyses”). Furthermore, we had closely examined all the MP according to the definition of contextual metaphor given by Forceville (2016). Also, we had separately conducted a step-by-step VISMIP, the visual metaphor identification procedure (Steen, 2018) and approved that all the MP had passed the test. The main steps of the procedure are listed below.

1. Look at the entire picture, to establish a general understanding of the meaning.
2. Structure the descriptive phrase(s). For example: “Smiling girl gives green apple to frowning boy under tree. [Agent(girl| smiling); Action(give); Object(apple| green); Recipient (boy| frowning); Setting(under tree).]”
3. Find incongruous visual units. (*For the present study, these must be “perceptually incongruous” and “conceptually incongruous”).
4. Test whether the incongruous units are to be integrated within the overall topical framework by means of some form of comparison.
5. Test whether the comparison(s) is/are cross-domain.
6. Test whether the comparison(s) can be seen as indirect discourse about the topic.
7. If the findings of steps 4, 5, 6 are positive, then a picture should be marked for metaphor.

*Adapted from: Instructions in VISMIP (Steen, 2018:82)*

Another 40 pictures were chosen from the same website, among which 36 were used as fillers and four as practice material. All the pictures chosen were relatively simple, clear, and easy to identify, with the same style, brightness, and size. Further, some pictures were edited by Adobe Photoshop 13.0 to remove the text labels if there were any from their originals, such as, information of the date, the title, the author’s signature, the logos, the watermarks, etc.

To ensure the validity and internal consistency of the three sets of experiment pictures, 90 students who did not participate in the experiment had been equally divided into six groups, and were directed to rate the pictures on a 5-point scale from six dimensions: metaphoricity, overall incongruency, perceptual incongruency, conceptual incongruency, complexity, and comprehension difficulty. Before the rating, all the participants were told that a pictorial metaphor is the visual representation of the verbal metaphor, and some sample pictures with familiar verbal counterparts were given as illustrations, e.g., TIME IS MONEY, BOOKS ARE STAIRCASES OF HUMAN PROGRESS, etc. Meanwhile, explanations were provided for a better understanding of the six dimensions. After the rating, on request, the participants reported that they had never seen any of the MP before. So the MP used in the present study can be deemed novel metaphors. The measurement for metaphoricity ranges from: 1 (literal) to 5 (metaphoric) (see Yang et al., 2013), for incongruency: 1 (congruous) to 5 (incongruous), for complexity: 1 (simple) to 5 (complex), and for comprehension difficulty: 1 (easy) to 5 (difficult).

As results of statistical testing showed, the scores of metaphoricity differ significantly, *F* = 546.92, *ps* < 0.001, MP (*M* = 4.22, *SD* = 1.01), ALP (*M* = 3.79, *SD* = 0.88), LP (*M* = 1.43, *SD* = 0.65). The scores of overall incongruency showed significant difference among the three conditions, *F* = 153.60, *p* < 0.01, MP (*M* = 3.87, *SD* = 1.11), ALP (*M* = 2.20, *SD* = 1.35), LP (*M* = 1.79, *SD* = 1.09). Both of the two subscales of incongruency displayed significant differences, respectively. Besides, within each subscale, the three conditions differed significantly one another, *ps* < 0.01: for perceptual incongruency, *F* = 615.68, *p* < 0.01, MP (*M* = 4.23, *SD* = 0.74), ALP (*M* = 3.83, *SD* = 0.80), LP (*M* = 1.69, *SD* = 0.66); for conceptual incongruency, *F* = 901.36, *p* < 0.01, MP (*M* = 4.40, *SD* = 0.58), ALP (*M* = 1.86, *SD* = 0.86), LP (*M* = 1.49, *SD* = 0.50). The scores of complexity displayed no significant difference across all conditions, *F* = 1.80, *p* = 0.17, with all scores below 2.5: MP (*M* = 2.25, *SD* = 0.62), ALP (*M* = 2.12, *SD* = 0.862), LP (*M* = 2.13, *SD* = 0.72). The scores of comprehension difficulty were all below 2.5 as well: MP (*M* = 2.32, *SD* = 1.32), ALP (*M* = 2.19, *SD* = 1.40), LP (*M* = 1.46, *SD* = 0.84). However, there existed significant differences in difficulty between MP and LP, ALP and LP, *ps* < 0.001. No significant difference was found between MP and ALP, *p* > 0.05. This result showed that although incongruous pictures were relatively more difficult to understand, the degrees of comprehension difficulty were below the average. The following is the within-rater reliability (Cronbach’s α < 0.05) of the six measures: metaphoricity (0.81), overall incongruency (0.84), perceptual incongruency (0.91), conceptual incongruency (0.92), complexity (0.88), and comprehension difficulty (0.90).

### 2.3 Procedure and data analyses

**FIGURE 1:**
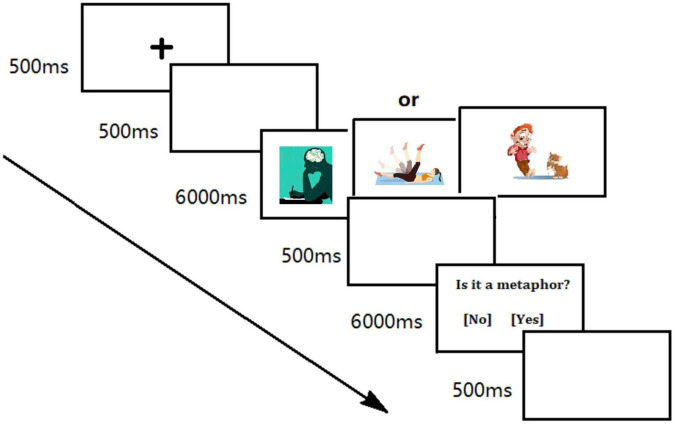
Experimental procedure.

An SR Research Eye-Link 1000 system was used with the sampling frequency of 1,000 Hz. The participants went through a 9-point calibration routine. A computer with 21-in. SVGA monitor was used to display the stimuli with a resolution of 1,024 × 768 pixels. The stimuli were 10 cm wide (8.60°) and 8 cm tall (7.05°). The participants viewed the screen from a distance of 65 cm. All the instructions and task specifications as well as the experimental procedure were given in Chinese. Before the experiments, the experimenter provided the definition of pictorial metaphors and some illustrations as in the rating section. Then the participants were instructed to accomplish a button-press task in which they were to decide whether a picture they had just viewed was a metaphor. Then the practice procedure began. Next, the experimenter gave feedback and communicated with the participant to ensure a complete understanding of the task. Then the experiment started (see Figure 1 for the procedure). At the beginning of each trial, a central fixation point “+” appeared and lasted for 500 ms in the center of the screen, followed by a blank screen lasting for another 500 ms. Then the picture appeared and remained for 6,000 ms. Eye movements were recorded during the picture browsing. When the picture disappeared, the metaphoricity decision task began. The participants had to judge as soon as possible whether the picture they had just seen was a metaphor or not. An answer must be given *via* a button press within 6,000 ms, then the trial ended with a 500 ms inter-stimulus interval. In a total of 72 trials, each picture was presented only once in random order. The experiment lasted about 30 mins.

**FIGURE 2:**
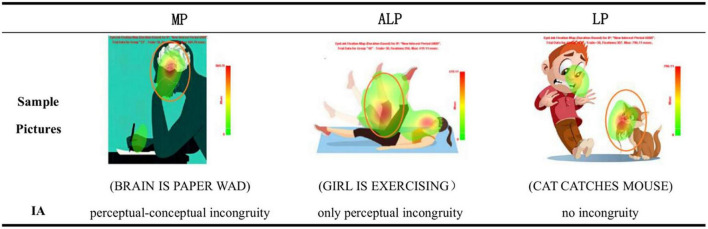
Sample output Fixation Maps of the three conditions with area of interest (IA) settings illustrated (Elliptical).

The area of interest (IA) of the picture was set as follows. For the MP, the IA was the contextual cue with perceptual-conceptual incongruity, i.e., the area where the target domain object had replaced the source domain object. For the ALP, the IA was the contextual cue with mere perceptual incongruity, i.e., the area where the representation of a target object was inconsistent with its real-life counterpart. As for the LP, to our knowledge, without a specific target or without incongruous (or anomalous) cues in the visual context, the area of interest may vary with different individuals. Although the IA could have been predicted by drawing results from our pilot experiment, we confirmed and finally established the IA for LP by referring to the output *Fixation Map*, which showed the density of duration-based fixations in a direct way (see Figure 2: All experimental materials will be available for readers on request made to the authors). The IA in each picture was sized at 37,000 pixels. The IA report was then exported to *Microsoft Excel* and the data were analyzed using SPSS 21.0.

## 3 Results and analysis

The invalid fixations with duration less than 80 ms were deleted from the data of 38 participants by following a cleaning procedure of the Eyelink Data Viewer, and then the data with two standard deviations above the RT mean in the metaphoricity decision task were removed (5.8%). A Single-Factor Repeated Measures ANOVA was performed. The Greenhouse–Geisser correction was used to adjust the *p*-values when the Mauchly’s test of sphericity had been violated, and the Bonferroni correction was applied in the *post-hoc* tests.

### 3.1 Behavior analysis

As could be expected, there is a decreasing gradient in the rating of the metaphoricity, with MP being the highest, followed by ALP and LP (see Table 1). The difference among the three conditions is significant, *F*(2, 74) = 159.15, *p* < 0.001, *η2p* = 0.81. The *post-hoc* test showed that significant differences in rating existed between MP and ALP, MP and LP, also between ALP and LP, *ps* < 0.001. This indicates that incongruous contextual cues had a great influence on MP comprehension, that is, the more abundant the incongruous contextual cues, the stronger the metaphor understanding. The statistical result of reaction time (RT) showed that there was significant difference among the three conditions, *F*(2,74) = 10.35, *p* < 0.001, *η2p* = 0.22. It’s significantly faster to judge MP than ALP or LP, *ps* < 0.01, but no significant difference was found between ALP and LP, *p* = 0.97. The RT results further showed that a picture was more likely to be identified as metaphorical when there were both perceptually and conceptually incongruous contextual cues. When the context was congruous or only perceptually incongruous, it took the participant more time to make a decision whether the picture was metaphorical or literal.

**TABLE 1:** Mean (SD) for metaphoricity rate and reaction time in different conditions.

	MP	ALP	LP
Rate	0.87 (0.15)	0.44 (0.19)	0.33 (0.21)
RT	1,180 (392)	1,400 (509)	1,380 (507)

### 3.2 Eye-tracking analysis

#### 3.2.1 Overall result in different conditions

Three eye-movement measures were selected to display the overall IA processing (see Table 2). *IA Fixation Percentage* refers to the percentage of all fixations in a trial falling in IA, representing the degree of interest given to the stimulus. *IA Fixation Count* refers to the total number of fixations in a trial falling in IA. The more counts means the more searches have been directed into an IA for the detail processing. *IA Dwell Time* represents the sum of the duration of all the fixations that fell in IA, showing the amount of attention and mental effort needed while processing the stimulus.

**TABLE 2:** Means (SD) for total area of interest (IA) fixations in different conditions.

	MP	ALP	LP
Fixation Percentage	0.65 (0.06)	0.56 (0.05)	0.54 (0.05)
Fixation count	11.84 (1.99)	10.01 (1.43)	9.69 (1.44)
Dwell time	3409 (402)	2931 (317)	2848 (361)

A repeated measures ANOVA was conducted and the result showed significant differences among all conditions in all of the three IA measures: fixation percentage, *F*(2,74) = 83.54, *p* < 0.001, *η2p* = 0.69; fixation count, *F*(2,74) = 64.44, *p* < 0.001, *η2p* = 0.64; dwell time, *F*(2,74) = 64.15, *p* < 0.001, *η2p* = 0.63. The *post-hoc* test showed that all of the three measures were much higher in MP than in ALP or LP, the differences being very significant, *ps* < 0.001. However, no significant differences were found between ALP and LP in all three measures, *ps* > 0.05. The results confirmed that it was more difficult to process MP than ALP or LP. This indicated an overall strong effect of perceptual-conceptual incongruity, whereas the effect of perceptual incongruity alone was not significant. Since no significant difference was found between ALP and LP, it could be inferred that the increased cognitive load in MP processing was simply caused by conceptual incongruity.

#### 3.2.2 The processing of contextual incongruity at different stages

The *IA Run Count* (the number of times the IA was visited) showed no significant differences among different conditions, *M* = 3.41, *SD* = 1.26, *F*(2,74) = 0.57, *p* > 0.05. Therefore, only three runs of IA fixations were selected for the comparative analyses (see Table 3 for the result).

**TABLE 3:** Means (SD) for area of interest (IA) fixations in different conditions at different stages.

		MP	ALP	LP
Start time	First run	280 (91)	244 (84)	336 (70)
Fixation count	First run	4.29 (1.29)	3.85 (0.91)	3.23 (0.93)
	Second run	4.05 (0.93)	3.04 (0.75)	3.08 (0.68)
	Last run	3.96 (1.21)	3.99 (1.20)	3.48 (1.16)
Dwell time	First run	1,201 (387)	1,111 (341)	891 (310)
	Second run	1,210 (306)	910 (229)	955 (239)
	Last run	1,254 (385)	1,267 (439)	1,135 (426)

*Area of interest (IA) First Run Start Time* reveals the start time of the very first fixating behavior. As Table 3 shows, the first run of IA processes started in the following sequence: ALP (244 ms), MP (280 ms), and LP (336 ms). The ANOVA result showed a significant difference among the three conditions, *F*(2,74) = 20.54, *p* < 0.001, *η2p* = 0.36. The *post-hoc* test showed that the IA processes started earlier in ALP and MP, whose IAs existed salient contextual incongruity cues, resulting in significant differences with LP, *ps* < 0.001. Whereas, there was no significant difference between ALP and MP, *p* > 0.05. *IA First Run Fixation Count* displayed a gradient from high to low: MP > ALP > LP, with a significant difference among all conditions, *F*(2,74) = 15.46, *p* < 0.001, *η2p* = 0.30. There were significantly more fixations in the two incongruous IAs (MP and ALP) than in the congruous IA (LP), *ps* < 0.01. However, no significant difference was found between MP and ALP, *p* > 0.05. *IA First Run Dwell Time* also formed a decreasing gradient: MP > ALP > LP, with a significant difference as well, *F*(2,74) = 14.06, *p* < 0.001, *η2p* = 0.28. The dwell time of the IAs with incongruous contextual cues (ALP and MP) were far longer than that with congruous cues (LP), *ps* < 0.01. No significant difference was found between the perceptual-conceptual incongruity condition (MP) and single perceptual incongruity condition (ALP), *p* > 0.05. Overall, it could be concluded that in the first run of IA processing, more fixations and longer duration as well as earlier fixating start time in both MP and ALP, were provoked by the perceptual incongruity.

In the second run of IA processing, more fixations and longer duration were found in MP than in ALP or LP, showing significant differences among all conditions in both measures: in fixation count, *F*(2,74) = 22.95, *η2p* = 0.38, and in dwell time, *F*(2,74) = 19.58, *p* < 0.001, *η2p* = 0.35. Whereas, no significant difference between ALP and LP was found in either fixation count or dwell time, *ps* > 0.05. The above result suggested that in the second run of IA processing, the effect of contextual cues with perceptual-only incongruity was not as robust as those with perceptual-conceptual incongruity. Meanwhile, it strongly proved that processing conceptual incongruity is substantially more difficult than processing perceptual incongruity.

In the last run of IA processing, the ANOVA result showed a significant difference among different conditions in fixation count, *F*(2,74) = 3.58, *p* = 0.033, *η2p* = 0.09. The *post-hoc* test revealed that although the IA fixations in MP were slightly more than that in ALP, the difference was not statistically significant, *p* = 0.054. Nor was there any significant difference observed in dwell time among different conditions, *F*(2,74) = 2.31, *p* > 0.05.

## 4 Discussion

### 4.1 The effect of contextual incongruity on pictorial metaphor comprehension

The behavior analysis showed that most of the participants were able to identify MP correctly. Compared with the congruous pictures, the incongruous pictures were more likely to be judged as metaphors. Perceptual-conceptual incongruity contextual cues even had a greater effect than the perceptual ones alone, for the former had significantly sped up the judgment. When posed with the two kinds of LP lacking salient conceptual incongruity cues, the individuals spent more time making decisions because such process would have probably involved extracting details from memory for evaluation and confirmation. Interestingly, compared to LP, ALP were more likely to be mistaken for metaphor. The higher false rate might be ascribed to the short time allowed for picture viewing and decision making, that is, only 6 s for each task. The incongruity in a certain ALP had been detected, and the residing perceptual incongruity might have well been solved by identifying the pictorial unit. Based on Relevance Theory (Sperber and Wilson, 1995), other things being equal, humans are naturally inclined to help each other and optimize the chance that their fellow beings understand them (Forceville, 2022). Since incongruity is a prerequisite, or “a signal” of metaphor (Shu, 2015:20), to the participants, the incongruity (anomaly) must have suggested something. However, time was not enough for the participants to further explore the possibility of alternative meaning beyond the literal meaning of the ALP. Consequently, the participants could only rely on the perceptual incongruity cues they had just managed to obtain and made a hasty decision, by overgeneralizing it that any anomalous looking pictures could be metaphors. That is in line with the previous studies that it’s hard for an individual to quickly abandon a metaphoric interpretation (Glucksberg and Keysar, 1990; Kazmerski et al., 2003). In contrast, the participants made positive judgment faster and more accurately in the condition of perceptual-conceptual incongruity. This result is consistent with previous studies (Ortiz et al., 2017; Cao et al., 2019), postulating that incongruity has a significant effect on metaphor processing (Romero and Soria, 2013; Shu, 2015).

The present study showed that the participants did strive to figure out a metaphor from an ALP, which supports the insight that metaphor does not necessarily build on a pre-existent similarity between A and B. In most of the cases, a metaphorical processing can create that similarity (Zhang and Forceville, 2020). The whole process of metaphor recognition must be challenging, dynamic (Gibbs and Santa Cruz, 2012) as well as effortful because “finding correspondences that look as if they are objectively there requires the construction of new imaginative meaning that is indisputably not there” (Fauconnier and Turner, 2002:20).

### 4.2 The trigger effect of perceptual incongruity on pictorial metaphor processing

The first run of IA processing in ALP (243 ms) and MP (280 ms) both started earlier than LP (339 ms), and no difference was found between the two incongruous conditions. This indicated that the processing of pictorial metaphor might first be triggered by the detection of the contextual cues of perceptual incongruity. According to the visual saliency framework (Henderson and Hollingworth, 1998), people’s initial eye movements during picture viewing are primarily controlled by visual features rather than by cognitive features. In the first 200 ms or so, the global features of the spatial layout will be automatically activated for scene recognition. In the meantime, the individual will pay special attention to incongruous regions and the contextual effect will come into play in the local information processing. Previous studies claimed that the process of discovering conceptual incongruity and further integrating it into the context should consume more cognitive resources (Coulson and van Petten, 2002; Steen, 2018). In the present study, there were no significant differences between MP and ALP either in fixation count or duration in the first visit to the incongruous IA, which strongly proved that in the 200–300 ms time window, it is the perceptual incongruity contextual cues that first capture the individual’s attention, rather than the conceptual incongruity induced by two conflicting cognitive domains of the metaphor.

Early studies on visual processing have shown that perceptual features are fundamental in the pictorial processing, be it in the whole process of object recognition (Loftus and Ginn, 1984) or before object recognition. Even unrecognized images can be pre-recognized only by their perceptual features (Snodgrass et al., 1996; Langley et al., 2008). At the pre-recognition stage when tackling the perceptual incongruity, metaphoric images as well as literal images could be seen and processed, but not yet recognized or understood. Furthermore, eye-tracking result of the present study supports the previous studies that neural activities caused by detecting and identifying the perceptual incongruity occur at the early stage of metaphor processing, as shown in such early ERP components as N270 (Cao et al., 2019), N300 (Federmeier and Kutas, 2002; Ma et al., 2016), or N400 (Barrett and Rugg, 1990; Ortiz et al., 2017).

### 4.3 The effect of incongruous contextual cues on the difficulty of processing

In the second run of IA processing, fixations, and duration in MP were both significantly more than those in ALP or LP, and no significant differences were found between the latter two conditions. The result suggested that it’s much more difficult to deal with the pictures with conceptual incongruity, which corresponds with the previous studies that, while perceptual representation plays a key role at the early stage of picture processing, conceptual representation plays a decisive role at the later stage (Snodgrass et al., 1996; Langley et al., 2008). According to Giora and Fein (1999), novel metaphors cannot be processed simply by retrieving knowledge from our memory, and the new meanings must be recalculated online. This involves making connections between different conceptual domains, and filtering out or suppressing unimportant features in course of selecting relevant conceptual domains, a process that requires considerable underlying neural resources. Further, the second processing of incongruous contextual cues in metaphoric interpretation involves additional cognitive processes, such as detection of semantic violation, semantic repair through cross-domain mapping, or categorization, semantic reanalysis, conceptual expansion (Abraham et al., 2021), etc. Those processes usually overlap with each other (Kazmerski et al., 2003), resulting in longer processing time. Therefore, even if there is sufficient contextual support to accelerate the conceptual integration, more cognitive efforts will still be needed when processing a picture in a metaphorical approach.

In the last run of IA processing, no substantial differences were found among various conditions. All the metaphors and the literals had similar fixation counts and duration. This suggested that the cognitive load may have been reduced at the completion of the perceptual and conceptual processing of contextual incongruity. The current result is in line with the finding of earlier studies on linguistic metaphor processing (e.g., Pynte et al., 1996), holding that the later stage of metaphor processing which mainly involved semantic integration, may well be in parallel with the literal processing.

## 5 Conclusion

The present study applied novel contextual pictorial metaphors in an eye-tracking experiment to explore the cognitive mechanism in metaphor processing. Apart from the incongruous MP and the baseline congruous literal pictures, we employed a midway condition—incongruous literal pictures. In so doing, the effect of contextual cues of conceptual incongruity could have been distinguished from that of perceptual incongruity. The major findings are summarized as follows.

First, incongruous contextual cues significantly influence pictorial metaphor comprehension. Pictures with contextual incongruity are more likely to be comprehended as metaphors. Incongruous contextual cues are facilitatory to the processing of metaphorical pictures, whereas these cues result in longer metaphor judgment on the literals. In the early stage, perceptual incongruity cues provoke the metaphor intentionality, while conceptual incongruity cues play a decisive role later in propelling the metaphorical mappings.

Second, the individuals are able to detect the anomaly in a picture within the first 200–300 ms, no matter the anomaly is induced by the perceptual-only incongruity or the perceptual-contextual incongruity. Similar early looking patterns on distinct incongruities reveal that the processing of pictorial metaphor may first be triggered by the detection of perceptual incongruity which mainly directs to attentional bias at the early stage. This finding resonates with the visual saliency theory.

Third, strong effect of metaphoric interpretation appears in the follow-up stage with more fixations and longer dwell time being recruited, reflecting greater difficulty, and heavier cognitive load when processing pictures with both perceptual and conceptual incongruity. This study provides eye-tracking evidence for the postulation that metaphorical processing consumes more mental effort than literal processing. However, both the congruous and the incongruous images are allocated with the same amount of cognitive resources in the final processing stage, supporting the idea that similar cognitive mechanism may function for both metaphorical and literal processing in the late time window when semantic integration is performed.

This eye-tracking study has shown how people understand metaphor in the visual form, and how metaphor helps the viewers to dissolve visual and cognitive conflicts to access the pictorial message. Contextual incongruity in pictures severs as the trigger of metaphor, which directs and changes the process of picture viewing, and further facilitates metaphorical mapping. In light of this, we assume that contextual incongruity in pictorial metaphor should be a varied and multidimensional thinking resource. This nature reflects its effect on the dynamics of cognitive evolution, which are “created through the constraints and affordances of the human brain, with its search for coherence and desire for novelty, and through the needs and pleasures of human social interaction” (Coulson, 2008:209). Moreover, our findings allow a sketch of an incongruity-based dynamic model for pictorial metaphor processing, where metaphor recognition and comprehension is a changing, dynamic and challenging process. At different stages of pictorial metaphor understanding, people try to form a dynamic coupling out of the apparently local incongruity in a larger global system composed of contextual factors, leading to metaphorical interpretation. We postulate that pictorial metaphor comprehension is not to recover a pre-existent meaning, but a tenacious quest for the optimal interpretation involving comparing, predicting, discovering, mapping, *ad hoc* category constructing, and verifying. During the conceptual expansion *via* the meaning-making processing, the incongruity-driven pictorial metaphor can inspire curiosity, insight, imagination, creativity, rather than state a visual fact.

This study has great implication in revealing the triggering and determining mechanisms of pictorial metaphor processing. These metaphor-unique mechanisms are significant in exploring human cognition and have great impacts on various facets of social and cultural communications. However, there are some limitations in this study, such as the relatively small amount of experiment material, and kind of subjectivity in designating the area of interest of the pictures. In addition, although the eye-tracking experiment has produced a large body of data resources, in this paper we’ve only discussed the effect of incongruous contextual cues on metaphor processing, which is just a glimpse at the whole picture. Future studies should make full use of the experimental methodology with good ecological validity apart from eye-tracking technique, for instance, ERP, real-time fMRI or fNIRS, etc., in the hope of finding other cognitive patterns in pictorial metaphor processing, for example, how can other contextual factors affect metaphor processing? Are there any different neural mechanisms underlying the processes of varied types of pictorial metaphor?

## Data availability statement

The original contributions presented in this study are included in the article/supplementary material, further inquiries can be directed to the corresponding author.

## Ethics statement

The studies involving human participants were reviewed and approved by Human Research Ethics Committee for Non-Clinical Faculties, the School of Psychology, South China Normal University. The participants provided their written informed consent to participate in this study.

## Author contributions

YM contributed greatly to this study and was responsible for conceptualization, methodology, software, experiment, formal analysis, and writing (original draft and editing). RZ was responsible for supervision, conceptualization, and writing. XC contributed to part of the software skill, experiment procedure, and data checking. All authors contributed to the article and approved the submitted version.

## Footnotes

1
https://www.dreamstime.com
